# Supplementary material for: Chromothripsis during telomere crisis is independent of NHEJ, and consistent with a replicative origin
Source: Genome Res. 2019 May;29(5):737–49. doi: 10.1101/gr.240705.118 (PMC6499312; doi:10.1101/gr.240705.118)
Supplement: Supplemental Material [file supp_gr.240705.118_Supplemental_file_1.zip › contigs/annotated_contigs/DB46/contig.3.DB46_length_844_mean_cov_4.95497630332.docx]

**DB46_length_844_mean_cov_4.95497630332**

```
CATATGAAATGTGGAGAGGCCCGTGTGGCAGAAGGAAAAGCAATGTGGAAGGTTTTAGGAACTTAGAGCCTCGATTCTACAGCTATAGG
 >chr20:10952076-10952315 - E=7e-132 p=4e-02
AAACTGAATTATGCCAACAACCAGAGTGAATTTGAAGCAGACCCCTCCCCAGTTGAAACTCCAGATAAAAGCACAGCCTAGCCAGTACT

GACTGGAGCCTGGTAAGACCCAGAAGCATAAAACCCAGCTAACTGTTACCTAGATTCCT|AA|ATGGTCATACCATTTACATGCCAAGC
                                                            >chr20:10950615-10950909 +
AGCCGTAAATCAGATTTTCAGTTTCCTCCACATTCTTGTCGAAACTTAATATTGACTATGGTTTTATTGTAGCCATTTTGGTGGGTAAG
E=6e-165
GCAAACCAAAAATACCATTAAAATCAGCACAGAAGTCCCCTTGCCCCAGTACTGATGTTTCATTGCATCTTAAGGAAACTTCTTTTATG

GGAGTAATCACCACAGGATGGATAGTTTAGTCCATATCTATAAAGGACTTGAAACAATTTAAAATGACTCACAGGGCAGCTGCATCAG|

CTGC|ATCCTTTTTCTGTGGTTATCTACCTTCTTTCTGATCACCTTCCTCATTTGTTGAAGACTTTGGCTCCTGGATCACAATATTCTT
    >chr20:51076764-51077073 + E=5e-171
TCTTCCTCCAGGTCAGACAACACTCTCAGAGATTTTGACATCTATGTTGAATTAGTGAGTGAGGGCCAGGGCTACTCACTATCCTGCCA

TGGGATACAGGGAATATAACGTAACAAAGGATTGTTTCACATCCTTGCATTTCTTCCTAGCATCCTGTTGGAAAGTCAGCTAGTTAAAA

AAACCTTATTATAATTATTTGGACCTAGAACCCAAATTCTATTTTAT
```
